# Multi-objective optimization of energy, view, daylight and thermal comfort for building’s fenestration and shading system in hot-humid climates

**DOI:** 10.1371/journal.pone.0325290

**Published:** 2025-06-18

**Authors:** Zhenling Wu, Yimin Xu, Zhuoyao Wang

**Affiliations:** 1 Center for Balance Architecture, Zhejiang University, Hangzhou, Zhejiang, China; 2 The Architectural Design and Research Institute of Zhejiang University Company Limited, Hangzhou, Zhejiang, China; 3 Department of Architecture, Hangzhou City University, Hangzhou, Zhejiang, China; 4 Zhejiang Engineering Research Center of Building’s Digital Carbon Neutral Technology, Hangzhou, Zhejiang, China; The Chinese University of Hong Kong, HONG KONG

## Abstract

Well-designed building envelope components are essential in addressing global warming. Fenestration and shading system (F&SS) not only promote energy conservation and emission reduction but also enhance occupant satisfaction by improving indoor environments. However, existing research often prioritizes energy use, daylight, and thermal comfort while neglecting view quality, a factor closely tied to mental health and productivity. This study employs multi-objective optimization (MOO) to balance energy consumption, view quality, daylight, and thermal comfort in office buildings located in hot-humid climates. By optimizing variables such as window-to-wall ratio (WWR) and shading device dimensions, the research integrates random forest models with SHapley Additive exPlanations (SHAP) analysis to quantify the influence of design parameters on optimization goals. Results indicate maximum improvements of 25.62% in energy use intensity (EUI), 23.18% in thermal comfort percentage (TCP), and 37.96% in useful daylight illuminance (UDI), highlighting the substantial potential of the proposed framework. This research refines the MOO framework for F&SS design, offering new insights into view quality considerations. Recommended values, such as a WWR of 0.6, provide practical guidance for architects in balancing energy efficiency and occupant comfort.

## Introduction

### Background

Human activities increase greenhouse gases in the atmosphere, which are considered the main cause of climate change [1,2]. The building sector significantly contributes to this issue [3], accounting for 34% of global energy demand and 37% of carbon emissions [4]. Well-designed building envelope components are crucial in combating global warming, as they can significantly improve buildings’ thermal performance [5,6]. F&SS regulates solar radiation entering a building [7], reducing cooling and heating energy consumption [8], while also improving occupants’ visual and thermal comfort [9,10]. Therefore, studying F&SS not only aids in energy conservation and emission reduction, but also enhance the health and well-being of building occupants by improving the indoor environment.

### Related work

Previous studies have identified several key facade-related parameters that influence energy performance and indoor environmental quality. These parameters can be categorized into four main groups: (1) Geometric parameters, such as window orientation (WFO) and WWR [11–15], which affect solar gain and daylight access. (2) The thermal performance parameters of building envelopes, particularly U-value and solar heat gain coefficient (SHGC) [16–18], directly determine their insulation and solar heat transmission, impacting energy efficiency. (3) Shading strategies, including external shading types, angle, depth, etc. [19–23], which mitigate heat gain and glare but may reduce visibility and daylight. (4) Ventilation related factors, such as openable window area ratio (OWR) [24–26] which contribute to natural ventilation and visual connection. In addition to their physical functions, facade and shading systems (F&SS) also significantly influence view quality, which is strongly linked to occupant health and psychological well-being [27]. However, optimizing view quality often conflicts with energy-saving and comfort goals. For example, shading systems reduce indoor temperatures and cooling energy demand by blocking solar radiation but also obstruct views [28]. Existing research has yet to adequately address this conflict. For instance, Huo et al. neglected view out, concluding that shading angles parallel to the window plane (i.e., full window obstruction) achieve optimal energy-saving performance [29]. Valitabar et al. considered view quality as a design reference, but evaluated it only in optimal models rather than as a variable in the analysis [30].

MOO has been widely applied in recent architectural studies to balance energy efficiency, visual and thermal performance [31,32]. For instance, Fan et al. proposed a multi-objective facade optimization method to enhance the visual environment and energy efficiency in stadium design [33]. This study demonstrates the applicability of MOO methods in public buildings, but it treats the curtain wall shading system as a whole, neglecting orientation factors‌. Nazari et al. proposed an optimization framework focusing on energy efficiency, visual, and thermal comfort, conducting a detailed analysis of factors such as shading component dimensions and angles [20]. However, the study was limited to two window shading configurations (e.g., south-facing horizontal shading). Wu et al. developed a MOO framework to optimize the building envelope design of south-facing rooms with horizontal shading device [26], without comparative studies on other orientations and shading devices. Existing studies have primarily focused on specific orientations and specific shading types, while systematic investigations that analyze multiple orientations and shading parameters remain limited.

Building optimization methods have advanced significantly, with genetic algorithms proving effective in finding high-performing design solutions [34–37]. Early-stage research employing Multi-Criteria Decision-Making (MCDM) [38] or Artificial Neural Networks (ANN) [39] for optimization, which relied on exhaustive simulations of all design combinations followed by time-consuming ranking processes. Alsharif et al. used an orthogonally structured training dataset to develop Ensemble Machine Learning (EML) models, incorporating the Multi-Objective Manta-Ray Foraging Optimizer (MOMRFO) to optimize shading devices [21]. However, the decoupled implementation of modeling and optimization processes across separate platforms introduced operational inefficiencies, highlighting the need for unified computational frameworks to streamline design iterations. Wallacei operates on the Rhino-Grasshopper platform, which integrates seamlessly with simulation plugins to enhance optimization efficiency and credibility. Liu et al. offered an optimization framework using the multi-objective optimization tool Wallacei based on the Non-dominated Sorting Genetic Algorithm II (NSGA-II) [40]. Hakimazari et al. focused on optimizing shading for an office room utilizing Wallacei’s algorithm and the Weighted Sum Method to find the optimal state [41]. The above studies validate the effectiveness of the MOO method using the Rhino-Grasshopper platform, offering a reliable reference for design decisions.

To sum up, recent relevant studies still have two main research gaps: 1) Although view out is important for occupants’ wellbeing and is strongly influenced by F&SS design, integrated analyses that consider view, energy, daylight, and thermal performance are relatively rare. 2) Existing studies are constrained by limitations in F&SS design variables, while in hot-humid climates, with low latitude and high solar radiation [27], shading system design requires more research [28].

### Research content

This study employed a genetic optimization algorithm to optimize the building’s F&SS parameters, aiming to minimize energy consumption, enhance visual and thermal comfort, and ensure adequate outdoor views for occupants. The objectives were measured using EUI (kWh/m2/y), TCP (%), UDI (%), spatial daylight autonomy (sDA) and view percent (VP). The design variables include WFO, WWR, OWR, shading device types (SDT), numbers (SDN), depths (SDD) and angles (SDA). After optimization, the interactions between various variables and objectives were analyzed by integrating random forest models with SHAP analysis, and optimal design recommendations were provided.

The major contributions and innovations are as follows:

- Developed a MOO system for F&SS to reduce energy use, enhance visual and thermal comfort, and improve view quality. By introducing VP as a quantitative indicator alongside EUI, TCP, UDI, and sDA, the framework ensures sufficient external views and improves the rationality of optimization outcomes.
- Expanded the independent variable system for F&SS in hot-humid climate zones. A wide range of external shading device parameters was included with detailed optimization designs for both vertical and horizontal shading forms.
- Integrated Machine Learning Analysis. This research integrates random forest models with SHAP analysis to quantify the influence of design parameters on optimization goals, aiding architects in informed decision-making.

This study provides architects with actionable recommendations for optimizing F&SS design in hot-humid climates, bridging existing research gaps and promoting holistic building performance improvements.

## Methods

### Research framework

**Fig 1:**
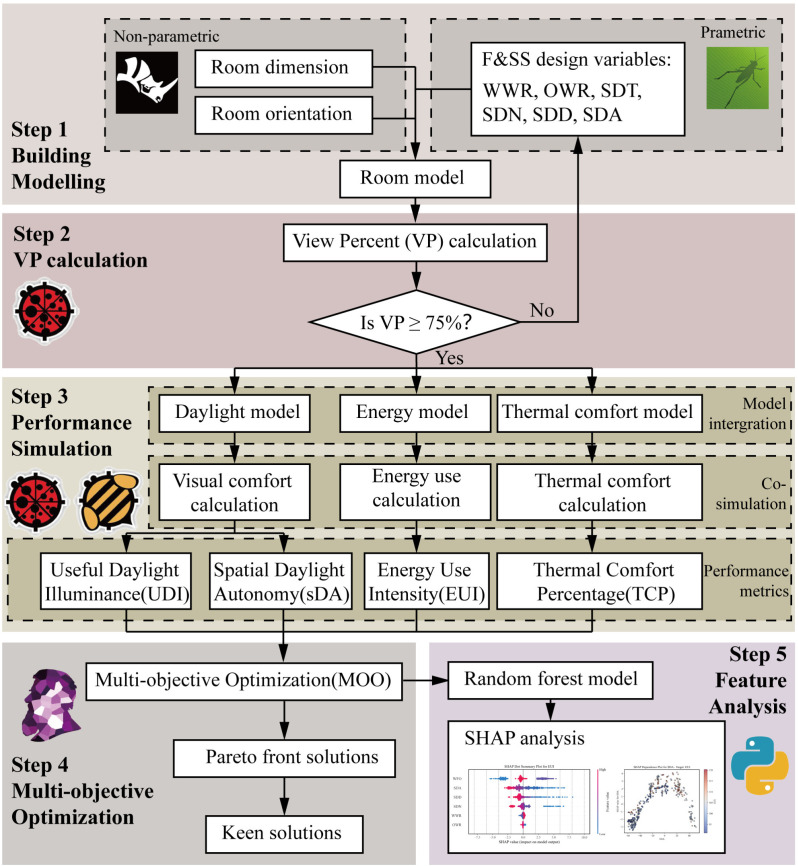
Optimization framework of office room.

The research framework includes five main steps, as shown in Fig 1.

1. Building modeling: Creation of the office room model in three orientations and parametric modeling of the F&SS design variables;
2. VP calculation: Evaluation of VP with a threshold criterion (VP ≥ 75%); models failing to meet this standard undergo iterative refinement;
3. Performance simulation: For each qualifying design, simulate and calculate the environmental performance of the room – including the energy performance, thermal and visual comforts;
4. Multi-objective Optimization: Using an evolutionary algorithm, the design space is searched to maximize UDI, sDA and TCP while minimizing EUI. Resulting Pareto fronts are subjected to preliminary statistical examination;
5. Feature analysis: A Random-Forest model, interpreted with SHAP values, quantifies the influence of each F&SS variable on the four optimization objectives.

This research framework used Rhinoceros 3D modeling software and its parametric plug-in Grasshopper for modeling. Based on the Rhino-Grasshopper platform, the Ladybug tools (2024) plug-ins were used for VP calculation and environment simulation, and the evolutionary engine Wallacei 2.7 was applied to perform MOO. Finally, the Random Forest algorithm coupled with SHAP analysis was applied to evaluate the contribution of each feature, using Python as the computing platform.

### The modelling process

#### Location and climate.

**Fig 2:**
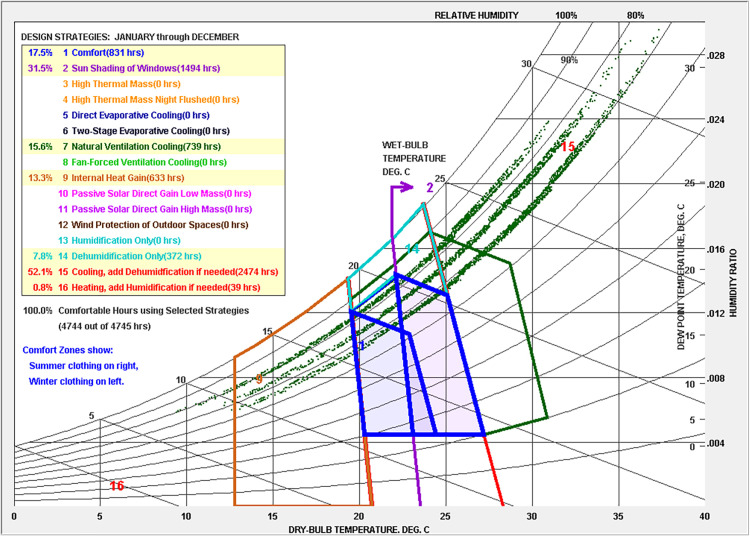
Psychrometric chart of Sanya.

Sanya (18°09’-18°37’ N, 108°56’-109°48’ E) is located in the south of Hainan Island in the South China Sea. It has a tropical ocean monsoon climate, with an average temperature of 25.4°C and 2563 hours of solar radiation annually [42]. The region experiences a long, hot, humid summer and a brief, mild winter [43,44]. The weather data was sourced from the Ladybug EPW map [45]. A psychrometric chart, created using Climate Consultant 6.0, outlines typical strategies for achieving comfort in Sanya (see Fig 2). Annual thermal comfort analysis revealed only 831 hours (17.5% of 4,745 total hours) met comfort criteria. Cooling and dehumidification requirements dominated 2,474 hours (52.1%), while shading strategies proved applicable for 1,491 hours (31.5%). Natural ventilation improved thermal comfort for 739 hours (15.6%), predominantly concentrated during November to March. The findings reveal the persistent challenges in thermal comfort under Sanya’s hot-humid climatic conditions, while validating the practical necessity of this research from a climate adaptation perspective.

#### The reference building.

**Fig 3:**
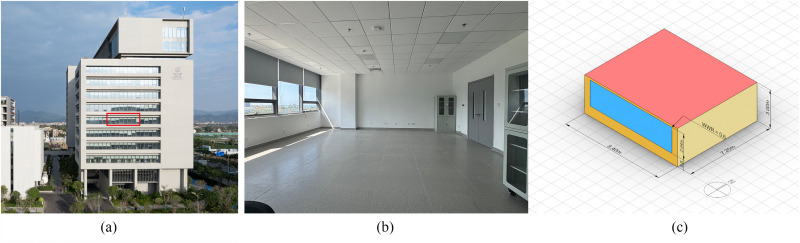
The case study room. (a) The exterior of the reference building; (b) The interior of the room; (c) The baseline model of the room.

The reference building is in Hainan Institute of Zhejiang University, Sanya, Hainan, China. A typical office room was chosen for the case study. It is an open office room on the middle floor with the dimensions of 7.2 m × 8.4 m × 3 m (length × width × height). It has one exterior wall with a window, while the other three walls are internal. The baseline model assumes a south-facing orientation, with a 7.4m × 2m (width × height) window in the middle (0.6 WWR) without external shading (see Fig 3).

**Fig 4:**
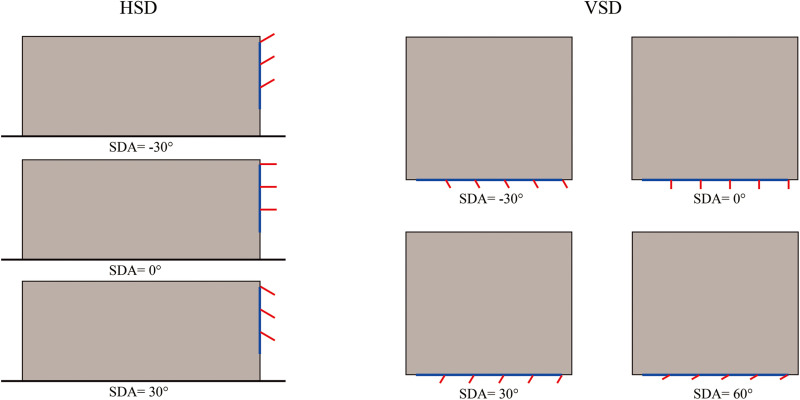
Examples of SDA for HSD and VSD.

This study primarily focuses on variables related to F&SS. The design parameters include WFO, WWR, OWR, SDT, SDN, SDD and SDA. The information and settings for these parameters in the Ladybug Tool are shown in Table 1. WFO considers the south, west, and east orientations. Changes in WWR were achieved by adjusting the window height, with the window width fixed at 7.4 m. SDT included Horizontal shading device (HSD) and Vertical shading device (VSD). SDNs for HSDs range from 1 to 5, while for VSDs they range from 1 to 10, with both types evenly distributed. When SDA is 0, the louvers are vertical to the window surface. For HSD, a positive SDA indicates the louvers tilt downward, while for VSD, a positive SDA means the louvers tilt to the left of the window (see Fig 4). Other variables, such as the material of the shading device, interior room material, and exterior wall U-value were fixed (see Table 2). Each office unit is assumed to accommodate 6 people during working hours (7 am – 7 pm).

**Table 1:** Building F&SS design parameters.

Parameters	Description	Baseline	Range
**P1: WFO**	Window facing orientation	South	[South 0°, West 90°, East 270°]
**P2: WWR**	Window-to-wall ratio	0.6	[0.2-0.6]
**P3: OWR**	Openable window area ratio	0.1	[0.1-0.5]
**P4: SDT**	Shading device types	None	Vertical(VSD)/Horizontal(HSD)
**P5: SDN**	Numbers of the shading device	None	Vertical: [1–10] Horizontal: [1–5]
**P6: SDD**	Depths of the shading device	None	[0.1m-1.0m]
**P7: SDA**	Angles of the shading device	None	[−60°-60°]

**Table 2:** Settings for the energy simulation model.

Parameters	Setting	
Weather data	The weather data were collected from the weather database in EPW files. (https://www.ladybug.tools/epwmap/).	
Building material data	Wall	U-value = 1.5 W/(m^2^·K)
	Window	U-value = 2.6 W/(m^2^·K), SHGC = 0.3, Material: Low-E glass, Transmittance:0.68
	Interior wall	Material: inorganic paint (matte white), Reflectance:0.75, Roughness:0.05
	Interior celling	Material: high crystal plate (light gray), Reflectance: 0.8, Roughness:0.05
	Interior floor	Material: ship-and-galley tile (light gray), Reflectance: 0.45,Roughness:0.05
	Shading device	Material: Concrete (gray), Reflectance: 0.32, Roughness:0.05
Internal loads	People	Occupant density: 0.1 people/m^2^ Activity schedule: 120 W/person
	Lighting	8 W/ m^2^
HVAC System	System type	DOAS with fan coil district chilled water with no heat
	Cooling set point	27 °C

The parametric configuration aligns with the technical specifications outlined in GB55015−2021, GB50176−2016 and GB50033−2013 [46–48].

### Building performance simulation

Ladybug Tools is an open-source plugin suite for Grasshopper that enables building performance simulation through engines like Radiance, EnergyPlus, and OpenStudio. The Ladybug module imports EPW weather data into Grasshopper and employs Continuous Daylight Modeling (CBDM) with Radiance for annual daylight analysis [49]. Its companion plugin Honeybee integrates Radiance, EnergyPlus, and OpenStudio simulations within Grasshopper, allowing users to define building parameters including geometry, materials, occupancy patterns, and equipment loads for comprehensive performance analysis. Widely applied in energy consumption, thermal environment, and daylighting simulations, Ladybug Tools has demonstrated its effectiveness through multiple research validations [26,37,40].

#### Visual comfort simulation.

The analysis of visual comfort performance was generally performed using computer simulation. Among the evaluation metrics, sDA and UDI are mostly used for evaluating daylighting conditions [37], these two metrics were also used in this research.

sDA describes the ratio of the space that achieves an ideal illuminance level for a particular amount of hours per year [50] (IES, 2012). It describes the overall factor of the daylighting performance with one single metric. For instance, sDA_300/50%_ refers to the percentage of the floor area where the illuminance exceeds 300 lx for at least 50% of the annual occupied hours (from 8 am to 5 pm) [51].


$$\begin{array}{c} \text{sDA}=\frac{\sum_{i=1}^{N}ST(i)}{N}\;with\;ST(i)=\left\{ \begin{array}{c} 1:{st}_{i}\geq {\tau t}_{y}\\ 0:{st}_{i}<{\tau t}_{y} \end{array}\right.\; \end{array}$$ (1)

Here, *st*_*i*_ represents the number of times the illuminance threshold for sDA is exceeded at point *i*; *N* is the total number of evaluation points; *t*_*y*_ is the total number of timestamps in a year, and *τ* denotes the temporal fraction threshold.

UDI is a widely-accepted paradigm intending to evaluate levels of daylight illuminance throughout an entire year [52]. Illuminances that fall within the range 100–2000 lx are considered useful, indicating the visual environment that is neither excessively bright nor dark [12].


$${UDI}_{100-2000}=\frac{\sum_{i=1}^{N}\alpha (i)\cdot t(i)}{\sum_{i=1}^{N}t(i)}\;with\;\alpha (i)=\left\{ \begin{array}{c} 1:100lx\leq I_{i}\leq 2000lx\\ 0:I_{i}<100lx\cup I_{i}>2000lx \end{array}\right.\;$$ (2)

Where *I*_*i*_ is the illuminance value of the *i*th point in the room; *t(i)* is the duration of the *i*th time interval, typically measured in hours; *α(i)* is a binary indicator that assigns a value of 1 when *I*_*i*_ lies within the useful range, and 0 otherwise; *N* is the total number of evaluation points.

Sensors were placed 0.75 meters above the ground, forming a grid of 0.8x0.8m [40]. The sensors obtained the percentage of the annual illuminance at each measurement point between 100 and 2000 lx, termed UDI_100–2000_. To assess the room’s overall illuminance, the average UDI_100–2000_ is used and referred to as UDI in this paper. The sDA_300/50%_ value, obtained by the same sensors, is referred to as sDA. Table 2 shows the optical properties of the building and windows, which are typical in China and comply with the Chinese daylighting design standards [48]. The design sky used in visual comfort simulation was created from weather data.

#### Energy simulation.

Winter in Sanya is short and mild, with an average temperature of 19°C. The ideal air system for this study used a fan coil district chilled water setup without heating, reflecting actual conditions. Previous research by Zhang et al. indicates that the 90% acceptable temperature range for hot-humid regions in China is between 23.5°C and 27.4°C [43]. Thus, the air conditioner’s cooling temperature was set to 27°C. The energy simulation output index is EUI in kWh/m²/year, which includes lighting power and cooling energy demands. Equipment load, being more related to room usage than F&SS, was not considered.


$$EUI=\frac{E_{Cooling}+E_{Lighting}}{A_{Room}}$$ (3)

*E*_*Cooling*_ is the total cooling energy (in kWh) obtained from the energy simulation; *E*_*Lighting*_ is calculated based on a constant lighting power density (8 W/m²) and operational hours; *A*_*Room*_ is the total area of the case study room.

#### Thermal comfort simulation.

Thermal comfort significantly affects occupants’ health and productivity [53]. In tropical climates, maintaining comfort requires considerable energy for cooling [54]. One design objective is to maximize comfort while minimizing energy use. This study used the adaptive comfort model derived from ASHRAE Standard 55 [55], aligning with the habits of people in hot-humid areas who rely on natural ventilation and adjust clothing seasonally [43]. Window opening behavior greatly influences the indoor thermal environment [25], and in this research, it was controlled by adjusting OWR. TCP was used as the indicator for measuring thermal comfort, representing the average percentage of time sensors recorded acceptable conditions during occupancy. Due to the fact that indoor thermal comfort in summer mainly relies on air conditioning for adjustment, this study focuses on the thermal comfort from November to March, considering the living habits of local residents.

#### View out simulation.

View quality has been assessed through many building grading systems, with LEED being the most widely used [28]. According to LEED v4.1 [56], 75% of the regularly occupied floor area should provide an exterior view. This study used the VP index to assess the overall quality of the view. VP represents the percentage of outdoor visibility from an interior space and can estimate the quality of a view from a given indoor location [57]. In this study, VP was calculated using Ladybug Tools 1.8.0, with measurement grids positioned at 1.1 meters above ground level. Unlike optimization objectives such as sDA, UDI, EUI, and TCP, VP was implemented as a threshold constraint during the optimization process. This decision stemmed from the significant conflicts observed between VP and building energy performance, thermal comfort, and daylighting metrics. Including VP as an optimization objective would have exacerbated inter-objective incoherence, thereby hindering algorithmic convergence. To balance visual quality with energy-environmental performance, a minimum VP threshold of 75% was established. All optimization solutions failing to meet this criterion were systematically discarded from consideration.


$$View\;Percent(VP)=\frac{Unblocked\;Rays}{Total\;Rays}\times 100\;\%$$ (4)

Where *Total Rays* are from a 360° horizontal band, limited above and below by a 30° offset from the horizontal plane, simulating human peripheral vision.

### Multi-objective optimization and data analysis

Given the conflicting objectives of energy efficiency, view quality, and visual and thermal performance, MOO was employed in this research. A four-objective optimization was conducted using Wallacei’s algorithm, based on the Grasshopper platform. Wallacei is primarily built on the NSGA-II [58], which iteratively evolves a population of solutions toward Pareto-optimality through selection, crossover, and mutation [59]. Six office room models -models with two different types of shading device (HSD or VSD) in three directions (south, west or east) – each underwent 30 iterations with a population size of 40. The mutation probability and crossover probability for the optimization algorithm were set at 1/40 and 0.9, respectively.

Within the Pareto front, solutions demonstrating optimal performance for specific objectives are referred to as extreme solutions, while solutions that balance trade-offs across all objectives are called knee solutions. The TOPSIS (Technique for Order Preference by Similarity to Ideal Solution) method was employed to identify these extreme and knee solutions.

For feature analysis, a Random Forest model was utilized alongside the SHAP method. Random Forest is a robust supervised machine learning algorithm widely applied to regression and classification tasks [60]. SHAP, based on Shapley values from game theory, has proven its effectiveness in revealing the relationships between input variables and prediction outcomes [61–63]. This method offers a valuable reference for design professionals when making informed decisions, facilitating the creation of architectural designs that better achieve intended results. The Python SHAP package was used for this analysis.

Feature analysis quantifies the influence of F&SS design parameters on optimization goals, enabling architects to comprehend the underlying relationships. Additionally, Knee solutions offer rapid and reliable predictive insights during the early stages of F&SS design in hot-humid climates, thus underscoring the practical value of this research.

## Results

### Pareto front

**Fig 5:**
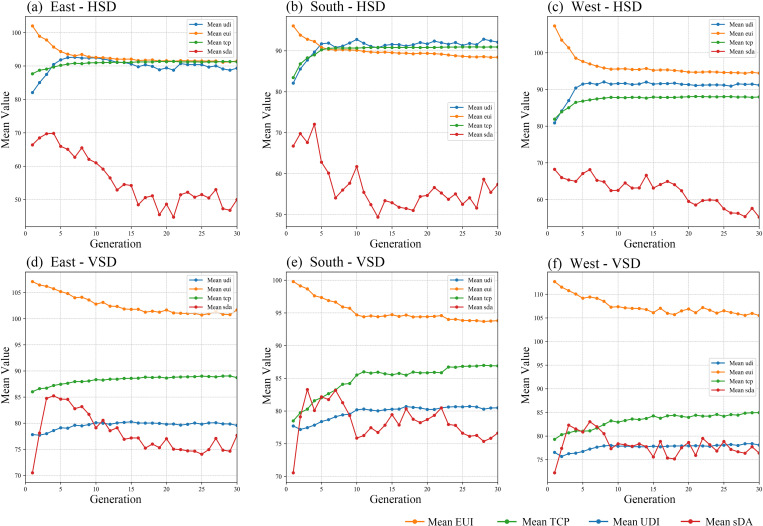
Trends of the mean values of targets.

The entire optimization process took approximately 240 hours. Fig 5 shows the trends of the mean values of targets for six models. As the iteration progresses, the EUI and sDA values in all models gradually decrease while the UDI and TCP values increase and stabilize. Energy use, visual and thermal comfort all improved.

**Fig 6:**
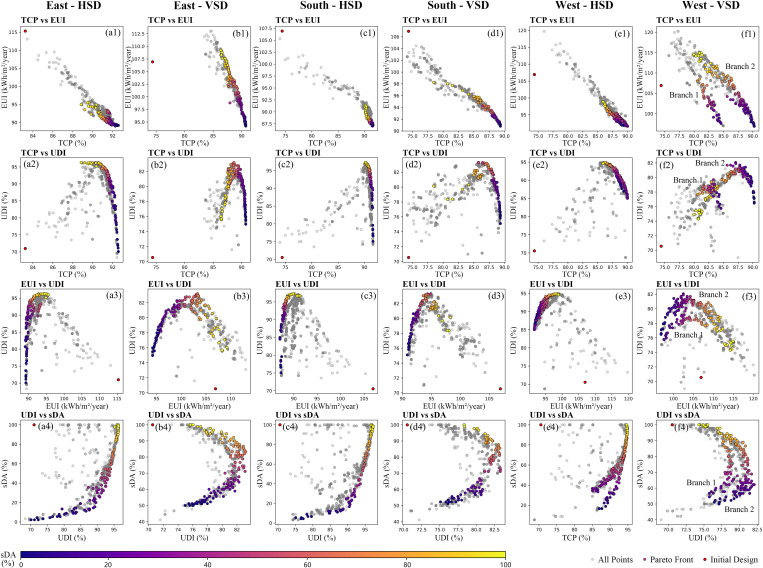
The 2D projections of Pareto frontier solutions and dominated solutions.

The optimization process generated a total of 7200 solutions, among which 662 were Pareto optimal solutions. Fig 6 illustrates the 2D projections of Pareto frontier solutions and dominated solutions of each model. Each gray dot represents a dominated solution, while the colored dots represent Pareto frontier solutions, with color variations indicating the size of the sDA value.

Fig 6a1–f1 show that in all models, the dominated solutions for TCP vs EUI exhibit a strong linear trend, with Pearson coefficients ranging from r = 0.707 to 0.977. The mean correlation is r = 0.907, indicating that higher thermal comfort is consistently associated with increased energy use across different orientations and shading types (see Table 3). TCP also shows moderate to strong negative correlation with sDA (r from –0.385 to –0.819), revealing a trade-off between thermal comfort and daylight autonomy. Moreover, a strong positive correlation between UDI and sDA is observed in most models with HSD (e.g., r = 0.67 in South – HSD; r = 0.695 in East – HSD), indicating a synergistic relationship between useful daylight availability and spatial daylight autonomy. However, this correlation diminishes or even reverses in configurations with VSD (e.g., r = –0.181 in East – VSD; r = –0.209 in West – VSD), implying that vertical shading may introduce spatial occlusions or glare control effects that decouple these two daylight metrics. These quantitative findings reinforce the visual trends illustrated in Fig 6 and enhance the robustness and clarity of the multi-objective trade-off analysis.

**Table 3:** Pearson correlation analysis between TCP, EUI, sDA and UDI in different orientations and shading devices.

	TCP vs EUI		TCP vs sDA		UDI vs sDA	
	r	p	r	p	r	p
**South – HSD**	0.885	< 0.001	–0.385	< 0.001	0.670	< 0.001
**South – VSD**	0.967	< 0.001	–0.664	< 0.001	0.172	< 0.001
**East – HSD**	0.945	< 0.001	–0.746	< 0.001	0.695	< 0.001
**East – VSD**	0.960	< 0.001	–0.819	< 0.001	–0.181	< 0.001
**West – HSD**	0.977	< 0.001	–0.696	< 0.001	0.545	< 0.001
**West – VSD**	0.707	< 0.001	–0.617	< 0.001	–0.209	< 0.001

All correlations are statistically significant at the 0.001 level (two-tailed).

Fig 6f1–f4 reveal that the Pareto front distribution for west-facing models with VSD differs significantly from other models, displaying two branches with similar trends, suggesting two distinct optimization directions. This situation is analyzed in detail in the discussion section.

### Extreme solutions

**Fig 7:**
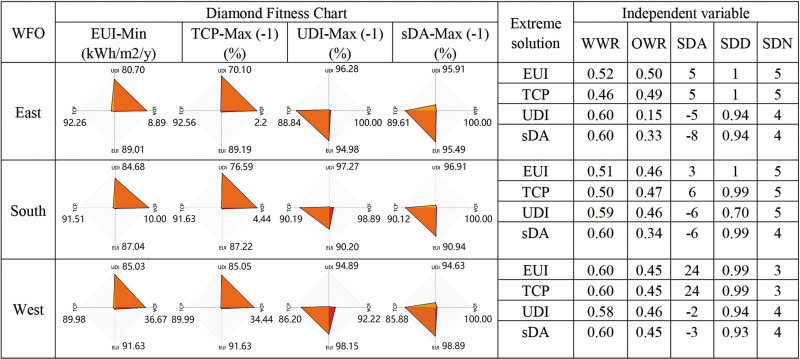
Summary of extreme solutions for all orientations – HSD.

**Fig 8:**
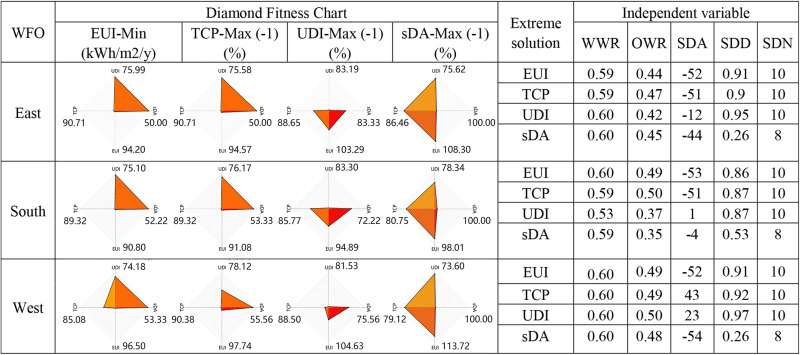
Summary of extreme solutions for all orientations – VSD.

Figs 7 and 8 summarize the objectives and parameters for each extreme solution corresponding to the four optimization objectives. These extreme solutions are identified from the Pareto front graph of each model.

The **best EUI** for rooms with HSD decreased by 18.60% to 25.62% compared to the baseline model, slightly surpassing the reduction observed with VSD. South-facing rooms achieved the lowest EUI, while west-facing rooms demonstrated the greatest energy-saving potential. Across all orientations, the best EUI solutions had improved UDI and TCP compared to the baseline, but sDA decreased significantly, with a reduction of 63.33% to 91.11% for HSD. This indicates that achieving the best EUI involves a substantial sacrifice in Daylight Autonomy. For the **best TCP** solutions, rooms with HSD improved by 11.21% to 23.18% relative to the baseline model, slightly exceeding improvements seen with VSD. South-facing rooms showed the greatest potential for enhancing thermal comfort. Similar to the best EUI, achieving the best TCP also results in a reduction of sDA.

The **best UDI** solutions for rooms with HSD improved by 35.66% to 37.96% compared to the baseline, which is twice the improvement seen with VSD. The UDI improvement potential is slightly greater in east and west orientations than in the south. The **best sDA** solutions reached the maximum value of 100 across all models, same as the baseline model.

### Knee solutions

**Fig 9:**
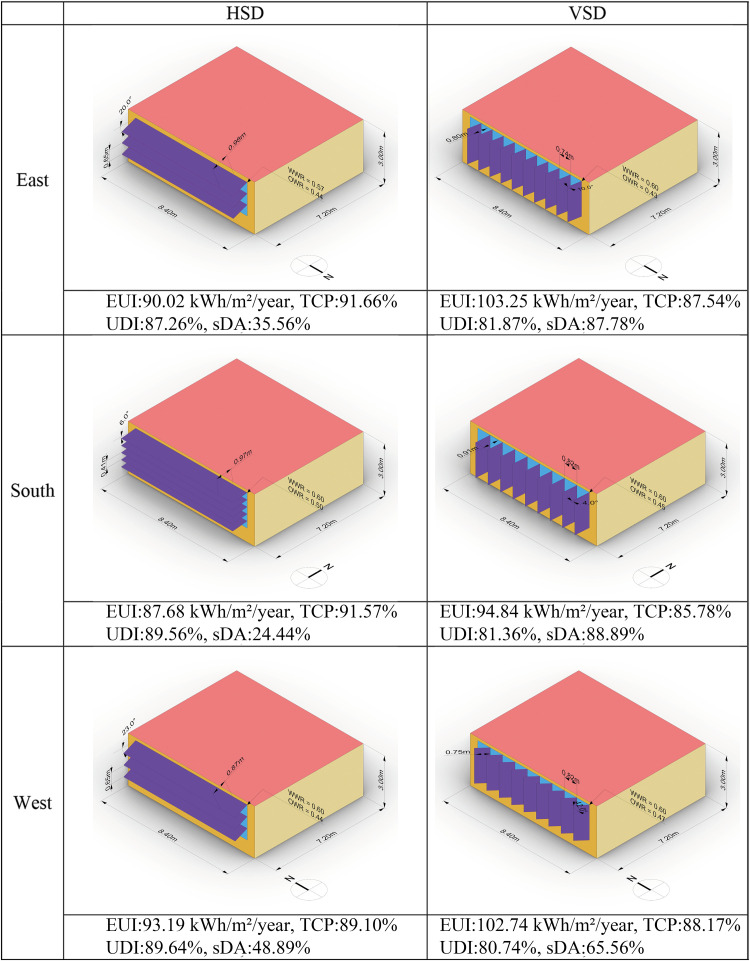
Knee solution for each model.

In the Pareto front solutions generated by Wallacei, six office room models with either HSD or VSD each underwent 1200 simulations in three directions. Each model produced approximately 100 Pareto front solutions. These solutions were then refined using the TOPSIS method, resulting in six comprehensive optimal solutions, as shown in Fig 9.

The average optimization for HSD in EUI, TCP, and UDI is 21.38%, 17.01%, and 26.74%, respectively. For VSD, the average optimization is 12.79%, 12.36%, and 16.02%. The average degradation in sDA for HSD is 63.70%, while for VSD it is 19.26%. Almost all models achieve a WWR of 0.6. SDA is lowest in the south orientation and highest in the west. HSD rooms prefer deeper SDD and a limited number of SDN, while VSD rooms tend to use relatively narrower SDD and a larger number of SDN.

### Parametric analysis with SHAP

**Fig 10:**
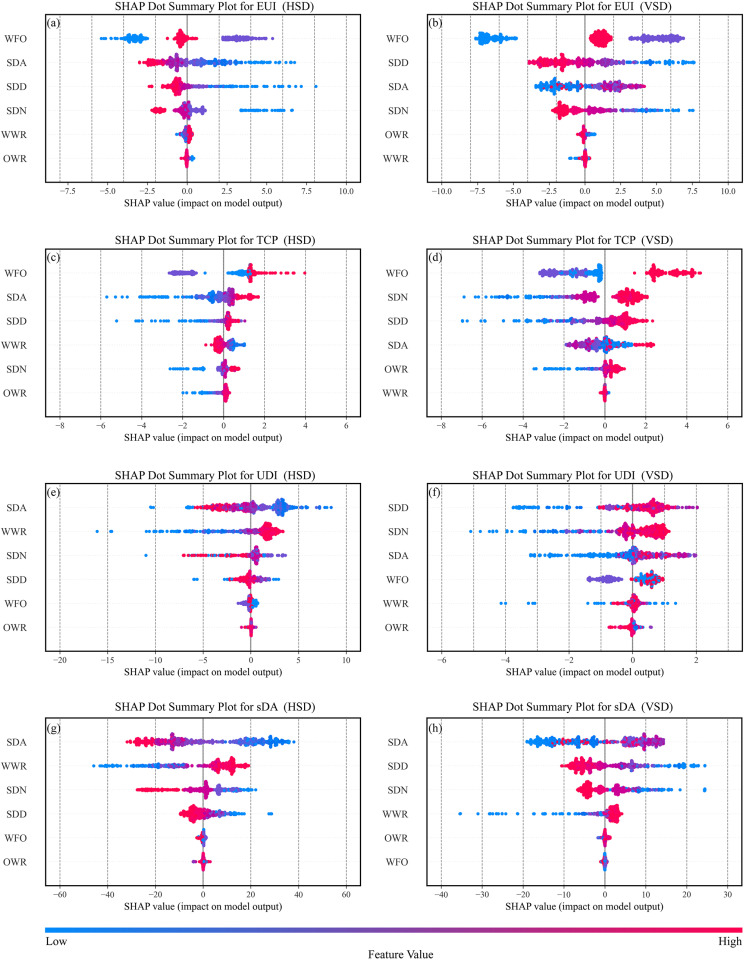
SHAP dot summary plot.

In this study, the SHAP summary plot was utilized to illustrate overall feature importance and understand how F&SS features influence the objectives. In Fig 10, the x-axis represents SHAP values, indicating the magnitude and direction of each feature’s impact on the objectives. The y-axis lists feature in descending order of importance. Each point on the plot corresponds to a specific dominated solution, with the color gradient transitioning from blue to red as the feature value increases. This red-to-blue distribution reflects the impact trend of each feature on the objectives.

For EUI, the most influential factor is WFO. Energy usage varies noticeably with orientation: south-facing (0°) rooms are the most energy-efficient, while west-facing (90°) rooms consume more energy. The second and third most significant factors are SDA and SDD. Generally, larger SDD, SDN, and OWR, combined with a smaller WWR, contribute to energy savings. For HSD, a larger SDA is preferable, while for VSD, a larger absolute value of SDA is more effective.

For TCP, east-facing rooms offer the best thermal comfort, while west-facing rooms perform the worst. Besides WFO, the key influencing factors for HSD rooms are SDN, SDD, and SDA, which align with the factors impacting EUI. For VSD rooms, the critical factors are SDA, SDD, and WWR. The variable impact trends on TCP closely resemble those on EUI.

For UDI, orientation plays a less significant role. For HSD, an upward tilt angle, larger WWR, and moderate SDN and SDD improve UDI. In contrast, VSD benefits from larger SDD and SDN with a moderate tilt angle to achieve higher UDI.

Regarding sDA, shading components consistently have a negative impact and are highly sensitive to SDA. Achieving greater sDA requires smaller SDD and SDN, larger WWR, and an upward tilt angle for HSD or an angle closer to the vertical plane for VSD.

## Discussion

### Pareto front

**Fig 11:**
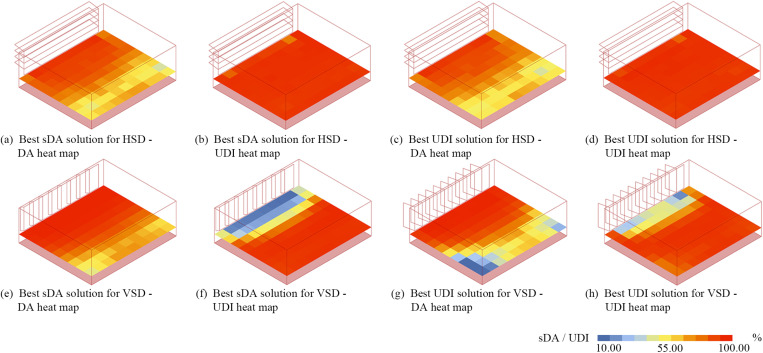
The UDI and sDA heat map for extreme solutions for east-facing room.

Energy consumption, thermal comfort, and visual comfort are mutually constrained. Fig 6a1–f1 shows a notable negative correlation between EUI and TCP, with Pearson coefficients ranging from r = 0.707 to 0.977, further supported by the SHAP dot summary plot in Fig 10a–d, where key factors influencing EUI and TCP are similarly ranked. This aligns with Nasrollahzadeh's findings on hot-humid climates [64] but contrasts with Wu et al. ‘s study on hot summer and cold winter regions [26], indicating the EUI-TCP relationship is climate-dependent. In hot-humid regions, buildings prioritize cooling with minimal or no heating in winter [65], whereas in hot summer and cold winter regions, energy-saving strategies vary significantly between seasons. Fig 6a3,c3,e3 reveals that EUI increases with UDI due to larger WWRs required to achieve higher UDI values. Larger WWRs allow more solar radiation, increasing cooling energy demand. This contrasts with Liu et al. 's study, where a light-sensing control system reduced artificial lighting demand, offsetting cooling loads and reducing overall EUI [40]. Daylighting metrics UDI and sDA also vary depending on shading design – horizontal (HSD) or vertical (VSD). Fig 6 a4-f4 shows that in HSD rooms, UDI increases with sDA, with Pearson coefficients ranging up to 0.695. While in VSD rooms, UDI initially rises with sDA before declining, with Pearson coefficients ranging from r = −0.209 to 0.172. This behavior occurs because sDA only specifies a minimum illuminance threshold, while UDI defines an acceptable illuminance range [66]. Shading devices improve UDI by reducing excessive lighting but may block natural light, decreasing sDA. Fig 11 highlights the heat maps of best sDA and UDI models under various shading strategies in east-facing rooms. With horizontal shading (Fig 11a–d), indoor daylight is more evenly distributed, and UDI and sDA maps align closely. In contrast, vertical shading (Fig 11e–h) shows lower UDI near windows in the best sDA models, suggesting glare issues. Compared to horizontal shading, vertical shading is more prone to glare problems [41], explaining the differing UDI-sDA patterns across the two shading strategies.

For west-facing rooms with VSD, optimization reveals two distinct branches (Fig 6f1–f4), which differ significantly from other models. Design parameter analysis shows that branch 2 solutions feature smaller SDN, SDD, and |SDA| values, prioritizing daylight autonomy. Branch 1 adopted an energy-efficiency-oriented strategy, intentionally compromising sDA by 17% to achieve 5% lower EUI. This unique scenario arises from intense low-angle solar radiation from the west [67]. Mitigating this issue requires larger SDN, SDD, and |SDA| louvers to block sunlight, which reduces daylight autonomy and leads to the two distinct optimization branches.

This study employed the VP index to evaluate room view quality, as F&SS primarily impacts the quantity and accessibility of external views [68]. However, using VP as an optimization objective may hinder convergence and negatively affect energy and thermal comfort optimization. According to LEED v4.1, 75% of the regularly occupied floor area should provide an exterior view to meet standards. Therefore, a minimum VP threshold of 75% was set, with results below this threshold deemed invalid. Key findings include: 1) HSD blocked views more than VSD, aligning with Alzoubi et al. ‘s findings [69]. HSD devices produced three times more invalid results during optimization. Taking the east-facing model as an example, the HSD model had 165 groups of invalid data, compared to only 48 groups for the VSD model. Additionally, HSD angles were generally closer to perpendicular to the window compared to VSD angles. 2) Optimal shading angles differ by view constraints. The optimal HSD angle was approximately 0° (perpendicular), consistent with De Luca et al.’s conclusion (2°) [70]. Without view considerations, angles of 30° [41] or 90° [29] were preferred. These differences highlight how view constraints push shading design toward maximizing exterior visibility, confirming their critical role in shading angle selection.

### The interactive effect between the variables and the objectives

This study employs SHAP Dependence Plots to investigate the complex relationships between variables and design objectives, offering insights into achieving better design outcomes.

**Fig 12:**
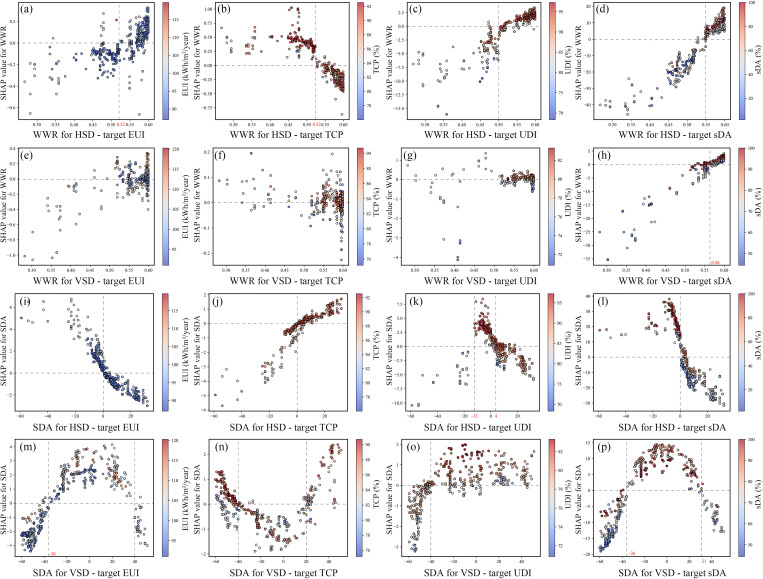
SHAP Dependence Plot for WWR and SDA.

The influence of WWR on each objective varies slightly depending on the shading configuration. Overall, reducing WWR benefits energy conservation and thermal comfort, but negatively affects the daylight environment. As shown in Fig 12a–h, for rooms with HSD, EUI decreases and TCP increases when WWR is below 0.52, while UDI and sDA improve when WWR exceeds 0.5 and 0.55, respectively. This indicates that the optimal WWR for HSD rooms is around 0.5. In VSD systems, the influence of WWR on each objective is less evident, but most optimal solutions converge around a WWR of 0.5–0.6, favoring larger WWRs. While larger WWRs negatively impact energy efficiency and thermal comfort, the results show most models adopt a WWR of 0.6 or close to it—much higher than the optimal WWR of 0.4 for hot climates proposed by Valladares-Rendón et al. [15]. The results suggest that shading components can effectively balance energy consumption, thermal comfort, and daylighting, achieving good overall performance even with large WWRs.

SDA plays a critical role in all models. As illustrated in Fig 12i–p, the relationship between SDA and design objectives in HSD rooms is linear: increasing SDA improves EUI and TCP but worsens UDI and sDA. In contrast, VSD rooms exhibit a parabolic relationship: moderate SDA values benefit daylighting, while extreme values favor energy efficiency and thermal comfort. The upward-tilted angle of HSD admits more natural light but also increases cooling demand due to solar radiation. The optimal HSD angle is around 0°. For VSD, angles between −36° and 31° are ideal for daylighting, while angles below −40° or above 40° prioritize energy conservation and thermal comfort. The lack of overlap between these ranges highlights a significant trade-off.

**Fig 13:**
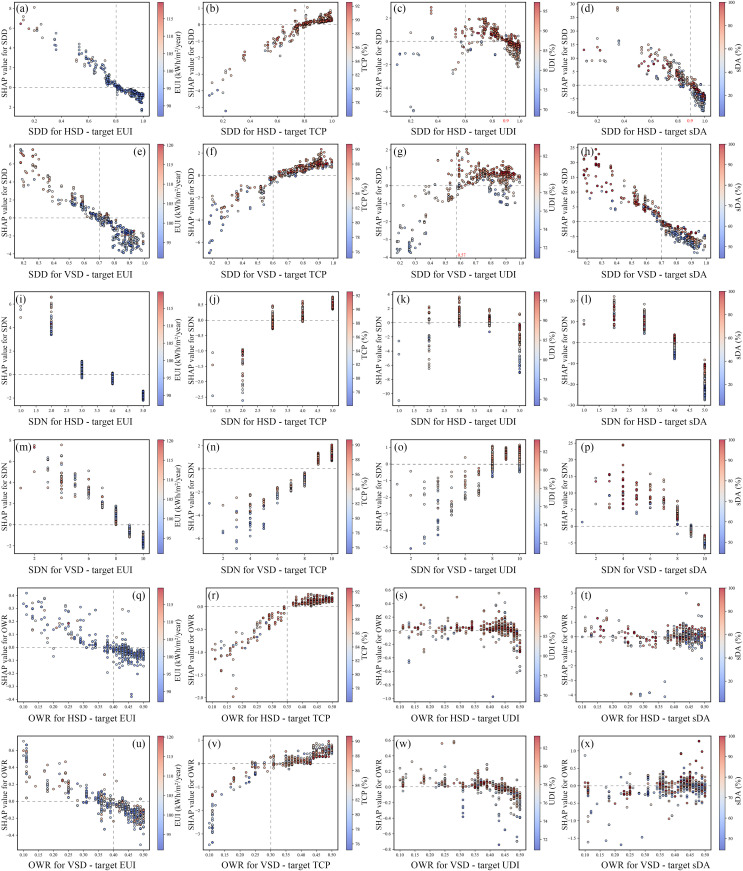
SHAP dependence plot for SDD, SDN and OWR.

Fig 13a–p demonstrates the influence of SDD and SDN on various objectives, with horizontal and vertical shading showing similar trends. Increasing SDN reduces energy consumption and improves indoor thermal comfort; however, further additions result in worse DA, consistent with Brzezicki et al.'s findings [71]. For HSD, the optimal SDD is between 0.8 and 0.9, with an optimal SDN of 4 and a louver spacing of 0.5 m. For VSD, the optimal SDD is approximately 0.7, with an optimal SDN of 9 and a louver spacing of 0.82 m.

OWR primarily impacts EUI and TCP, showing a notable trend regardless of shading type (see Fig 13q–x). An OWR greater than 0.4 is preferred, indicating that increasing OWR has a positive influence on energy efficiency and thermal comfort, aligning with the findings of Mao et al. [25]. Therefore, window opening design should be a key consideration in façade design.

### The recommended ranges for design variables

Based on Fig 9 and Figs 12–13, the recommended ranges for design variables are summarized as follows: **1) WWR**: The optimal range is 0.5–0.6. **2) SDA**: For south-facing rooms, the optimal SDA is around 0° for both HSD and VSD. For east-facing rooms, the optimal angle is 20° for HSD and 10° for VSD. For west-facing rooms, the optimal angle is approximately 20° for HSD, while VSD requires a larger angle of around 40°. **3) SDD**: The optimal depth for HSD is around 0.9, with smaller depths recommended for west-facing orientations (0.87) and larger depths for south (0.97) and east-facing orientations (0.98). For VSD, the ideal depth is 0.7–0.8, with larger depths recommended for south-facing orientations (0.91) and smaller depths for east (0.8) and west-facing orientations (0.75). **4) SDN**: For HSD, the optimal SDN is 4, with a spacing of 0.51 m. For VSD, the optimal SDN is 9, with a spacing of 0.82 m. **5) OWR**: The recommended range is 0.4–0.5.

For west-facing orientations, large SDA (40°) with small SDD (0.87) are preferred due to the lower solar altitude, which increases the risk of glare [67]. A larger SDA effectively blocks direct sunlight, while a smaller SDD balances daylighting performance. Adjustments to the SDN and SDD of shading devices can further optimize daylighting and energy efficiency. The recommended SDN falls within the top 20% of the variation range, indicating a preference for more devices with shallower depths over fewer devices with greater depths. This ensures uniform indoor daylight distribution and reduces thermal radiation, contributing to energy savings.

Existing national energy efficiency design standards [47,72] provide general recommendations for selecting shading devices based on building orientations and specify requirements for the shading coefficient, which is defined as the ratio of solar radiation received at windows with and without external shading. However, these standards lack detailed guidelines for specific shading design measures such as shading device dimensions and angles. The proposed shading design strategies in this study offer practical guidance for architectural design in hot-humid climate zones.

Another notable observation in knee solutions is that, for rooms facing the same orientation, HSD generally outperforms VSD in terms of EUI, UDI, and TCP, but results in sDA value below 50%. This aligns with Alzoubi et al.’s findings, which suggest that HSD rooms have lower overall brightness compared to VSD rooms [69]. Consequently, HSD rooms may require increased artificial lighting, highlighting a potential trade-off that warrants further investigation.

### Limitations and future research directions

Increasing WWR enhances indoor lighting, thereby reducing lighting energy consumption and partially offsetting cooling loads. In hot-humid climates, the additional direct solar radiation admitted through large windows can raise mean-radiant and operative temperatures, thus impairing thermal comfort even when cooling is provided. However, this study applies a static lighting load of 8 W/m² without considering its relationship to illuminance levels, and solar-heat-gain-induced discomfort was not explicitly modelled. These simplifications may misestimate energy use and comfort, leading to conservative design guidance. Future research should consider integrating lighting conditions with energy consumption and balancing this with potential thermal discomfort from solar radiation (e.g., incorporating spatial-temporal metrics to evaluate direct solar exposure risks near windows).

In hot-humid climates, opening windows during the transitional season enhances indoor ventilation and heat exchange. Window dimensions and shading device positioning affect airflow environment, subsequently influencing indoor thermal comfort [73]. In this study, we focused on OWR to simplify analysis, considering model design and simulation constraints. It also does not account for the impact of wind on F&SS, despite the high wind speeds and frequent typhoons in Hainan’s coastal cities, which can significantly affect external shading systems. Future work is suggested to evaluate the impact of wind to enhance indoor ventilation efficiency while reinforcing the wind resistance of shading louvers, accommodating typhoon conditions and reducing maintenance costs.

The analysis of view quality in this study remains preliminary. Due to optimization convergence limitations, VP was adopted as a threshold criterion rather than an optimization target. This approach prevented an intuitive understanding of how ‌F&SS design variables‌ influence view quality. Future research directions should prioritize incorporating VP as a target parameter and analyzing view quality through compositional metrics (e.g., view content and depth) to further refine shading system optimization frameworks.

## Conclusions

This study employed a MOO approach to improve the F&SS design of office buildings in China’s hot-humid climate region. By adjusting WFO, WWR, SDT, SDN, SDD, SDA, and OWR, the study aimed to achieve a coupled optimization of energy consumption, thermal comfort, and visual comfort while meeting view-out requirements. The key findings are as follows:

- **Optimization Results:** Rational F&SS design achieved maximum optimization rates of 25.62% for EUI, 23.18% for TCP, and 37.96% for UDI compared to initial values. EUI and TCP exhibited consistent optimization trends, but trade-offs emerged with daylighting, with Pearson coefficients ranging from r = –0.385 to –0.819 between TCP and sDA.
- **Parametric analysis**: For EUI and TCP, a lower WWR (below 0.5) combined with higher shading parameters - SDA (30° for HSD and absolute value above 40° for VSD), SDD (0.9-1.0), SDN (8-10) - significantly improves EUI and TCP, but reduces sDA. Conversely, UDI benefits from a higher WWR (0.5–0.6) with moderate shading dimensions, such as SDA (around 0°), SDD (0.6–0.9), and SDN (3 for HSD and 9 for VSD).
- **Recommended Parameter Ranges**: A WWR of 0.6, OWR of 0.4–0.5, SDD of 0.75–0.98, and a moderate number of SDN (4 for HSD and 9 for VSD) contribute to optimal comprehensive performance. SDA is highly influenced by orientation, with south-facing rooms requiring angles near 0°, west-facing rooms the largest angles (HSD 20°, VSD 40°), and east-facing rooms falling in between (HSD 10°, VSD 20°).
- **SDT Recommendations**: HSD outperforms VSD for EUI, TCP, and UDI but results in 50% lower sDA. Designs prioritizing energy efficiency and thermal comfort should favor HSD, while those targeting better daylighting and DA performance should prefer VSD. HSD parameters should feature larger SDD (0.9–1.0) combined with a moderate SDN (spaced at 0.5 m), while VSD parameters should adopt moderate SDD (0.7–0.8) with a greater SDN (spaced at 0.8 m) to achieve optimal overall performance.

## Supporting information

S1 Dataset Simulation data for office room models. (XLSX)

## Abbreviation:

EUI: energy use intensity
SDN: numbers of the shading device
F&SS: fenestration and shading system
SDT: shading device types
HSD: horizontal shading device
SHAP: SHapley Additive exPlanations
MOO: multi-objective optimization
TCP: thermal comfort percentage
NSGA-II: Non-dominated Sorting Genetic Algorithm II
UDI: useful daylight illuminance
OWR: openable window area ratio
VP: view percent
SDA: angles of the shading device
VSD: vertical shading device
sDA: spatial daylight autonomy
WFO: window facing orientation
SDD: depths of the shading device
WWR: window-to-wall ratio
